# Histological Subtype Provides Limited Prognostic Information in Grade 1 Invasive Breast Carcinoma: A Retrospective Cohort Study

**DOI:** 10.3390/cancers18193154

**Published:** 2026-09-29

**Authors:** Eleonore Locher, Lara Benning, Zsuzsanna Varga

**Affiliations:** 1 Department of Pathology and Molecular Pathology, University Hospital Zurich, 8091 Zurich, Switzerland; 2 Internal Medicine, See-Spital Horgen, 8810 Horgen, Switzerland; 3 Department of Pulmonology, University Hospital Zurich, 8091 Zurich, Switzerland

**Keywords:** breast cancer, histological grade 1, prognostic relevance

## Abstract

**Background:** Histological grade is one of the strongest prognostic factors in invasive breast cancer. However, it remains unclear whether histological subtype provides additional prognostic information in well-differentiated (grade 1; G1) invasive breast carcinomas. This study investigated the distribution of histological subtypes in G1 breast cancers and evaluated their impact on overall survival. **Methods:** In this retrospective single-centre study, all patients diagnosed with G1 invasive breast carcinoma in a single institution between 2005 and 2014 were identified. Histopathological and clinical data were retrieved from institutional pathology and clinical databases. Tumours were classified as no special type (NST), pure special type, or mixed subtype. Overall survival was analysed using Kaplan–Meier estimates and compared with the log-rank test. Associations between histological subtype and mortality were evaluated using the chi-squared test. **Results:** Of 429 initially identified patients, 364 fulfilled the inclusion criteria. NST represented the predominant subtype (67.3%), whereas pure special types and mixed subtypes accounted for 15.1% and 15.7% of cases, respectively. Tubular carcinoma was the most frequent pure special subtype, followed by mucinous, cribriform, papillary, lobular and adenoid cystic carcinoma. After exclusion of patients with simultaneous higher-grade contralateral carcinomas, 348 patients were included in survival analyses. The overall 5- and 10-year survival rates were 94.7% and 85.4%, respectively. Pure special types showed the highest survival rates numerically, whereas mixed subtypes had the lowest. However, overall survival did not differ significantly between NST, pure special types and mixed subtypes (log-rank *p* = 0.39). Likewise, no significant survival differences were observed among individual pure special subtypes (*p* = 0.20), and no significant association between histological subtype and mortality was identified (χ^2^ *p* = 0.064). **Conclusions:** Within this cohort of G1 invasive breast carcinomas, no statistically significant association between histological subtype and overall survival was identified. However, clinically relevant differences cannot be excluded given the heterogeneity of the cohort and the small number of events. Larger studies incorporating multivariable analyses and breast cancer-specific outcomes are needed to further clarify the prognostic relevance of histological subtype in G1 invasive breast cancer.

## Simple Summary

Breast cancers are classified according to their histological grade and subtype, both of which provide information about tumour characteristics and patient prognosis. Grade 1 breast cancers are well differentiated and generally have a favourable outcome. However, it is unclear whether different histological subtypes within this low-grade group are associated with different outcomes. We studied 364 patients with grade 1 invasive breast cancer diagnosed at a single institution. Most tumours were invasive breast carcinomas of no special type, while approximately one third represented pure special types or mixed subtypes. Survival was generally excellent, with overall survival rates of approximately 95% at 5 years and 85% at 10 years. Although patients with pure special tumour types showed numerically better survival than those with mixed subtypes, these differences were not statistically significant. Larger studies are needed to determine whether histological subtype provides additional prognostic information in patients with grade 1 breast cancer.

## 1. Introduction

Breast cancer is the most common non-cutaneous malignancy in women worldwide and comprises a biologically heterogeneous group of tumours with distinct morphological, molecular and clinical characteristics [1]. Patient prognosis is determined by a combination of clinicopathological parameters, including tumour stage, lymph node status, biomarker profile, histological grade and histological subtype [1,2,3,4,5]. Among these, histological grade according to the Nottingham Grading System remains one of the most robust and reproducible prognostic factors and is routinely incorporated into therapeutic decision-making [2,4,5,6,7,8].

Approximately 75% of invasive breast carcinomas are classified as invasive carcinoma of no special type (NST), whereas the remaining tumours comprise a heterogeneous group of histological special types as defined by the World Health Organization (WHO) [2,6,9]. According to the recently updated WHO Classification of Breast Tumours (6th edition), histological subtype remains an integral component of breast cancer classification [2]. However, its independent prognostic value remains uncertain once established prognostic variables are taken into account [1,2,10,11]. Several of these special types, including tubular, mucinous and cribriform carcinoma, are generally associated with an excellent clinical outcome, whereas others, such as high grade metaplastic carcinoma, show a more aggressive biological behaviour [1,2,6,9,10,11,12,13,14,15]. However, interpretation of these observations is complicated by the fact that histological subtype is closely associated with other favourable prognostic variables, including lower histological grade, smaller tumour size and lower nodal stage [2,4,5,7,16,17,18,19].

Previous studies investigating the prognostic value of histological subtype have produced conflicting results [11,14,17,20]. While several large cohort studies demonstrated differences in outcome between individual special types, others concluded that histological subtype alone provides only limited prognostic information once established prognostic variables are considered [11,14,17,20]. In particular, histological grade appears to outweigh subtype as an independent predictor of survival [14,18,20]. Consequently, it remains uncertain whether the favourable prognosis reported for many special histological types reflects their intrinsic biology or simply their frequent association with low-grade disease [21,22,23]. In our previous study, we investigated the prognostic relevance of pure and mixed special histological subtypes in a cohort that included carcinomas of different grades [14]. In this study, we focused specifically on the potential prognostic power within the G1 group.

To address this question, studies investigating homogeneous patient cohorts are required. Restricting analyses to grade 1 carcinomas minimises one of the strongest confounding prognostic variables and allows a more direct assessment of the potential prognostic contribution of histological subtype. Despite the clinical relevance of this question, data focusing exclusively on well-differentiated invasive breast carcinomas remain scarce.

The aim of the present study was therefore (1) to determine the distribution of histological subtypes in grade 1 invasive breast carcinomas diagnosed at a large tertiary referral centre and (2) to evaluate whether histological subtype is associated with overall survival in this prognostically favourable subgroup.

## 2. Materials and Methods

### 2.1. Study Design and Patient Cohort

This retrospective single-centre cohort study included consecutive adult patients diagnosed with grade 1 (G1) invasive breast carcinoma at the Department of Pathology and Molecular Pathology, University Hospital Zurich, between 1 January 2005 and 31 December 2014. Cases were identified from the pathology database of the Pathology Institution (PathoPro, version 9.1.0.8678) using the search terms *breast* and *G1*.

Clinical and pathological data were retrieved from the pathology database and complemented by review of the electronic medical records (KISIM, version 5.6.0.14). Collected variables included patient demographics, biopsy and surgical pathology reports, histological subtype, Nottingham grade, tumour stage, lymph node status, receptor status (ER, PR and HER2), lymphovascular invasion, surgical treatment, adjuvant therapy, clinical events and follow-up. The most recent documented patient contact was used for survival analyses. The study formed part of a larger breast cancer project approved by the Ethics Committee of the Canton of Zurich (KEK-2012-553; PB-2019-00111, BASEC 2024-01804).

### 2.2. Histopathological Assessment

Only patients with a final diagnosis of grade 1 invasive breast carcinoma in the surgical specimen were included. Cases upgraded to grade 2 or grade 3 following surgical resection were excluded. Cases downgraded from grade 1 invasive carcinoma on core biopsy to ductal carcinoma in situ (DCIS) at excision remained eligible, and the biopsy subtype was retained for descriptive analyses.

Tumours were classified according to the current WHO criteria (4th, 5th and 6th editions of the WHO classification of breast tumours) as invasive carcinoma of no special type (NST), pure special histological type, or mixed subtype. Mixed carcinomas were defined as tumours containing more than one histological subtype, irrespective of the relative proportions of the individual components.

### 2.3. Statistical Analysis

Descriptive statistics were used to summarise clinicopathological characteristics and the distribution of histological subtypes. Patients with simultaneous contralateral invasive carcinomas of higher histological grade were excluded from survival analyses because prognosis is determined by the highest-grade tumour.

Overall survival was calculated from the date of diagnosis to death or last follow-up using the Kaplan–Meier method. Patients alive at the last documented follow-up were censored. Owing to the limited number of events, median survival was not reached; therefore, 5- and 10-year overall survival rates were reported. Survival distributions were compared using the log-rank test. However, overall survival was not specifically related to breast cancer-specific death.

The distribution of follow-up duration among histological groups was assessed using the Kruskal–Wallis test after confirmation of non-normality with the Kolmogorov–Smirnov and Shapiro–Wilk tests. Associations between histological subtype and mortality were analysed using the chi-squared test. Statistical analyses were performed with IBM SPSS Statistics version 30.0. A two-sided *p*-value < 0.05 was considered statistically significant.

## 3. Results

### 3.1. Patient Characteristics

**Figure 1:**
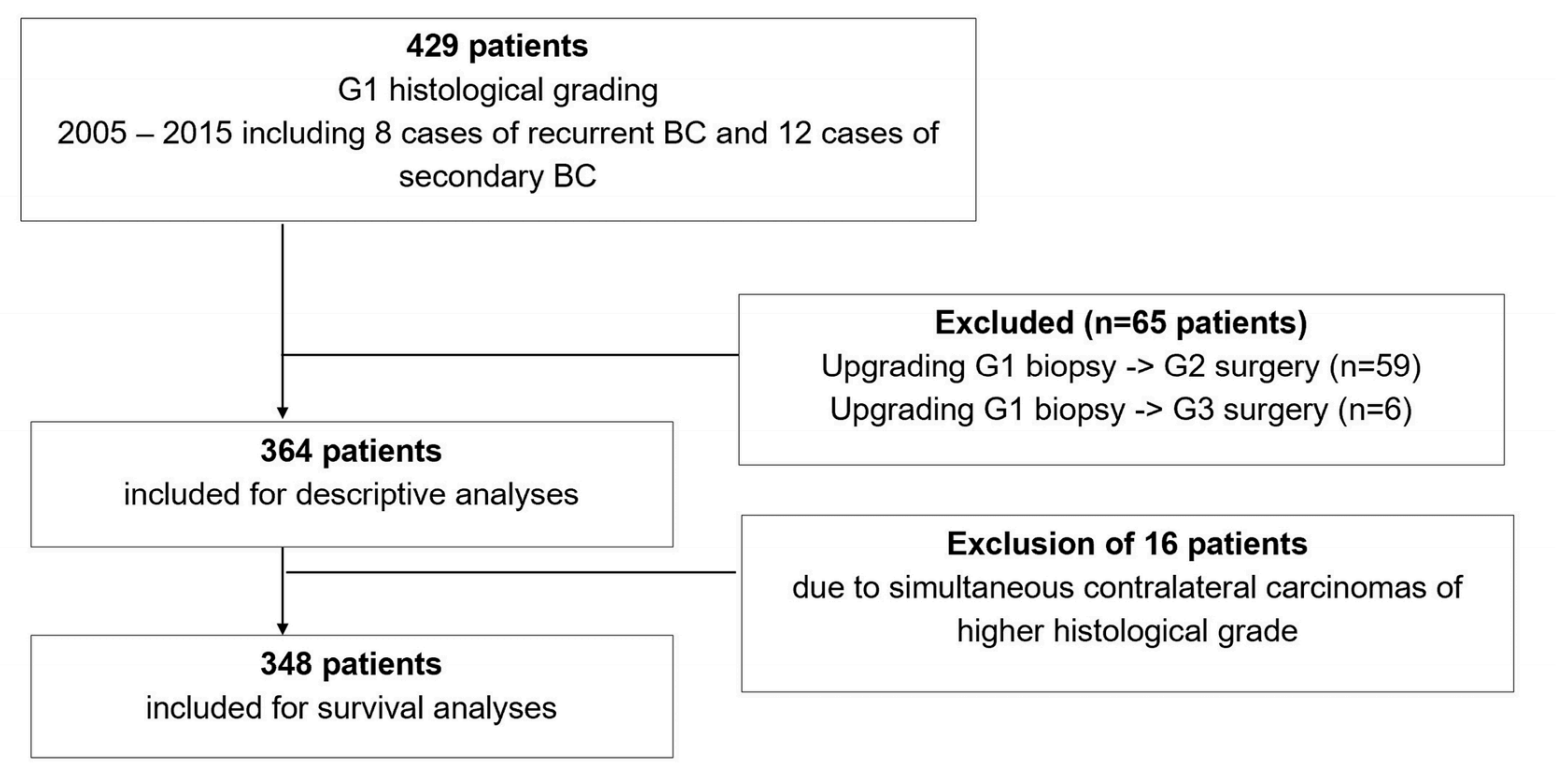
Chart-flow of case selection (exclusion and inclusion of patients). BC: breast cancer.

**Figure 2:**
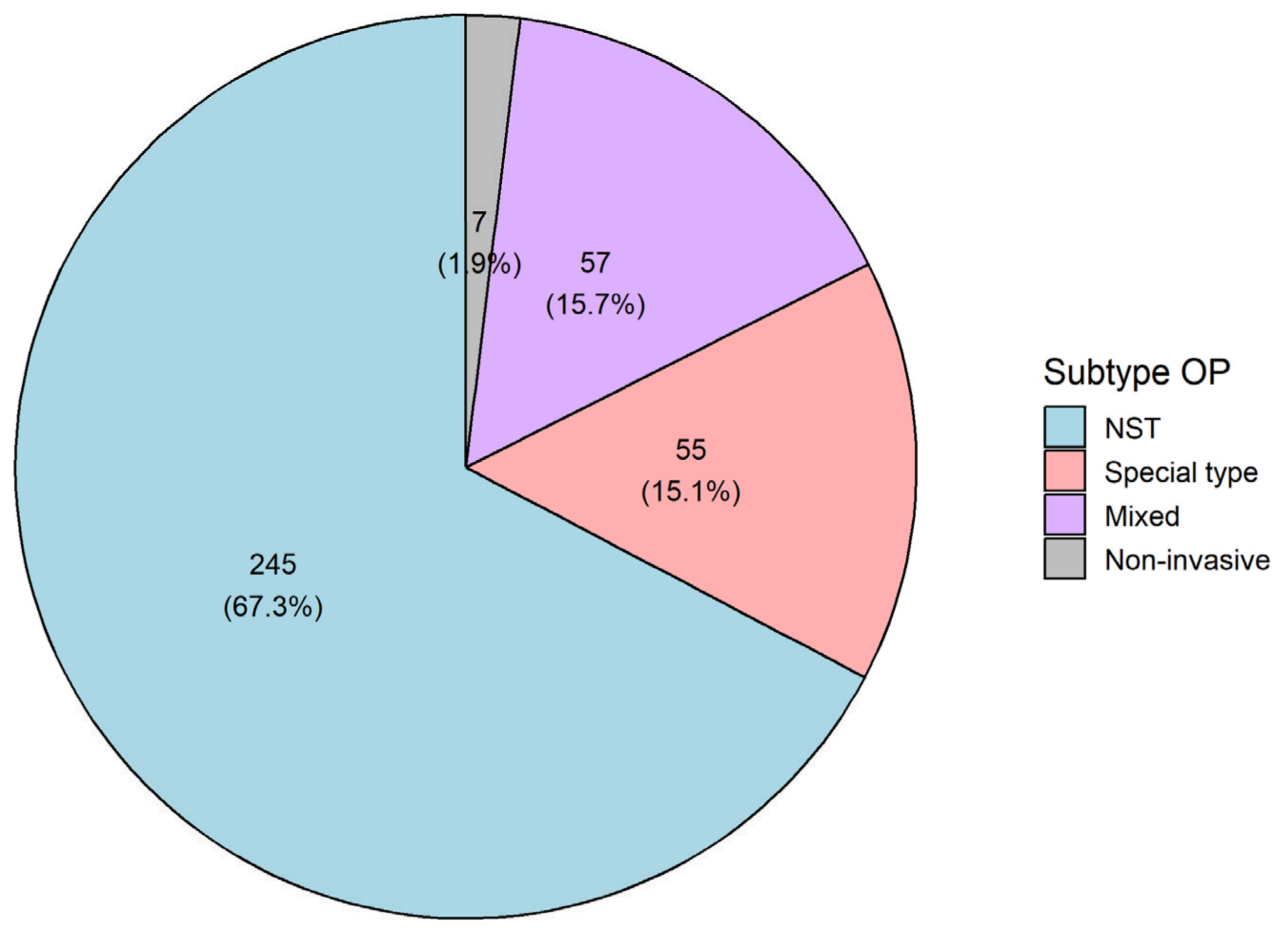
Overall frequency of histological subtypes of G1 carcinomas in the whole cohort. OP: Operation.

A total of 429 patients with grade 1 breast carcinoma were initially identified. Following exclusion of 65 tumours that were upgraded to grade 2 or grade 3 in the surgical specimen, 364 patients were included in the final study cohort (Figure 1). The median age at diagnosis was 62 years (range, 33–92 years), and all patients were female (Figure 2).

Histological grade differed between core biopsy and surgical resection in 85 cases (19.8%). Upgrading from grade 1 at biopsy to grade 2 or grade 3 at surgical excision occurred in 65 patients (15.2%), whereas 18 tumours initially diagnosed as grade 2 on biopsy were downgraded to grade 1. In 90 patients (24.7%), no core biopsy was available for review.

Details are shown in Table 1 and Table 2. The number of surgeries per year is shown in Supplementary Table S1.

### 3.2. Distribution of Histological Subtypes

Among the 364 surgical specimens, invasive carcinoma of no special type (NST) represented the predominant histological subtype (245/364, 67.3%). Pure special histological types and mixed carcinomas each accounted for 57 cases (15.7%) (Figure 2 and Figure S1).

Mixed carcinomas comprised 16 different histological combinations, most commonly NST with cribriform differentiation, NST with tubular differentiation, and NST with combined cribriform and tubular differentiation, together accounting for more than 60% of all mixed tumours (Table 3).

### 3.3. Survival Analysis

After exclusion of 16 patients with synchronous contralateral higher-grade carcinomas, 348 patients were included in the survival analyses.

Overall survival for the entire cohort was excellent, with 5- and 10-year survival rates of 94.7% and 85.4%, respectively. During follow-up, 46 patients (13.2%) died, whereas 302 (86.8%) were censored (Supplementary Table S2).

**Figure 3:**
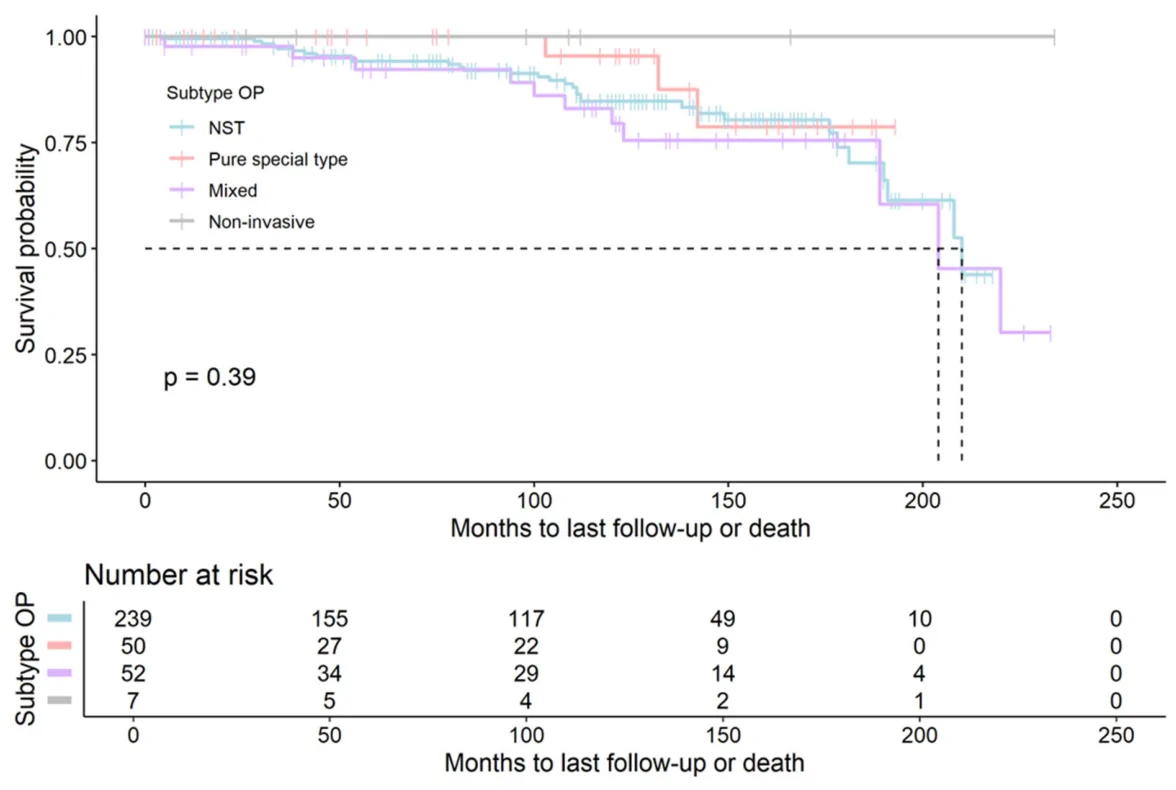
Kaplan–Meier survival curves in subtypes of G1 carcinomas (NST vs. pure special type vs. mixed subtypes) in surgical specimens in a follow-up period of 20 years. OP: Operation. There is no significant difference in overall survival stratified by histological subtype (*p* = 0.39).

When stratified by histological category, pure special types demonstrated the highest survival rates (5-year: 100%; 10-year: 95.7%), whereas mixed carcinomas showed numerically lower survival (5-year: 92.1%; 10-year: 79.5%). Patients with NST had survival rates comparable to those of the overall cohort. However, Kaplan–Meier analysis demonstrated no statistically significant difference in overall survival among NST, pure special types and mixed carcinomas (log-rank *p* = 0.39) (Figure 3).

Within the pure special-type group, only three deaths occurred during follow-up, affecting patients with cribriform, mucinous and adenoid cystic carcinoma. No deaths were observed in patients with tubular, papillary or lobular carcinoma. Survival differences between individual special histological subtypes were not statistically significant (log-rank *p* = 0.2) (Supplementary Figure S2).

Because mixed carcinomas comprised 16 distinct subtype combinations, many represented only one to three cases, precluding meaningful statistical comparison between individual combinations. The available dataset in this cohort does not contain the cause-of-death and longitudinal event data required for reliable analyses of these endpoints. The 46 deaths therefore represent deaths from all causes and cannot be attributed solely to breast cancer.

### 3.4. Follow-Up and Mortality Analyses

Follow-up duration did not differ significantly between the three principal histological groups (Kruskal–Wallis *p* = 0.412), confirming comparable observation periods.

Similarly, no significant association was observed between histological subtype and mortality (χ^2^ = 5.50, *p* = 0.064; Cramer’s V = 0.126), indicating only a weak relationship between histological subtype and overall survival.

## 4. Discussion

The present study investigated whether histological subtype provides independent prognostic information in a homogeneous cohort of patients with well-differentiated (grade 1) invasive breast carcinoma. Although numerical differences in survival were observed between histological groups, histological subtype could not be significantly associated with overall survival. These findings suggest that, within grade 1 invasive breast carcinomas, the favourable prognosis is predominantly determined by low histological grade rather than by histological subtype. However, the small number of deaths, particularly among patients with special histological subtypes, limited statistical power. No statistically significant association between histological subtype and overall survival was identified in this cohort, while clinically relevant differences cannot be excluded.

An additional noteworthy observation was the relatively high frequency of grading discordance between core biopsy and surgical excision, occurring in approximately one-fifth of cases.

Previous studies have consistently demonstrated prognostic differences between individual histological subtypes of breast carcinoma [14,18,19,20]. Tubular, mucinous and cribriform carcinomas are generally associated with excellent outcomes, whereas high grade metaplastic carcinoma and several other rare subtypes exhibit more aggressive biological behaviour [2,5,10,12,13,18,24,25]. However, most published series included tumours of all histological grades and stages. Consequently, the reported prognostic differences may, at least in part, reflect the unequal distribution of established prognostic variables rather than the independent effect of histological subtype itself [2,5,10,12,13,18,24,25].

By restricting our analysis to grade 1 carcinomas, one of the strongest prognostic determinants was largely controlled. Within this biologically favourable subgroup, histological subtype no longer demonstrated a statistically significant influence on survival. This observation is consistent with previous studies suggesting that histological grade carries greater prognostic weight than histological subtype and that subtype should be interpreted in conjunction with other clinicopathological parameters rather than in isolation [2,5,10,11,12,13,18,24,25,26].

Interestingly, patients with pure special histological types showed numerically better survival than those with NST or mixed carcinomas. Although this trend did not reach statistical significance, it is biologically plausible and in keeping with the recognised favourable behaviour of several special histological subtypes [2,5,10,11,12,13,18,24,25,26]. The absence of statistical significance is likely attributable to the relatively small number of events and the limited sample size within individual subtype categories, particularly among the rare tumour entities.

An additional finding of this study was the relatively high frequency of grading discrepancies between core needle biopsy and the corresponding surgical specimen. Approximately one-fifth of cases demonstrated a change in Nottingham grade, with most representing upgrading at surgical excision. Although not the primary objective of the present study, this observation underlines the well-recognised limitations of tumour grading on core biopsy and may warrant further investigation in larger contemporary cohorts.

### Strengths and Limitations of the Study

The strengths of this study include the uniform pathological assessment at a single tertiary referral centre, the homogeneous study population restricted to grade 1 invasive breast carcinomas and the long duration of clinical follow-up, approaching 20 years in some patients. These factors reduce diagnostic variability and allow robust assessment of long-term outcome.

An important further strength of the present study is the separate analysis of mixed histological carcinomas. In routine practice, these tumours are frequently grouped according to their predominant component and are therefore underrepresented in outcome studies. Their separate evaluation provides additional insight into this clinically relevant but insufficiently studied subgroup.

Several limitations should also be acknowledged. The retrospective design inevitably introduces selection bias, while treatment modalities evolved during the study period and were not incorporated into the survival analyses. Furthermore, the relatively small number of deaths limited statistical power, particularly for analyses of individual special histological subtypes and mixed carcinomas. Consequently, small prognostic differences cannot be completely excluded.

Although treatment modalities were not incorporated into the survival analyses and evolved during the study period, all patients were managed within a single tertiary breast centre using multidisciplinary treatment recommendations, thereby reducing interinstitutional variability.

## 5. Conclusions

In conclusion, this study demonstrates that histological subtype alone could not be associated with significant prognostic power in grade 1 invasive breast carcinoma. These findings reinforce the central prognostic role of histological grade while supporting the continued diagnostic importance of histological subtype for tumour classification and multidisciplinary patient management. Larger multicentre studies incorporating contemporary treatment strategies are warranted to validate these observations.

Our findings do not diminish the diagnostic importance of histological subtype. Histological classification remains essential for accurate tumour diagnosis, recognition of rare entities, correlation with characteristic molecular alterations and appropriate multidisciplinary management. Rather, our results suggest that, within the specific subgroup of well-differentiated invasive breast carcinomas, histological subtype contributes only limited additional prognostic information beyond histological grade. The retrospective design introduces potential selection bias. Treatment approaches also changed during the study period and were not accounted for in the survival analyses. In addition, the small number of deaths limited statistical power, particularly for analyses of individual special histological subtypes and mixed carcinomas. Small prognostic differences therefore cannot be excluded.

## Figures and Tables

**Table 1:** Patients with G1 histological grading in biopsy (*n* = 429).

	***n* (%)**
**Patients’ characteristics**	
Sex	
Female	429 (100)
Male	0 (0)
Age at diagnosis (years)	
median (IQR; range)	61 (51–71; 33 to 92)
≤50	91 (21.2)
>50	338 (78.8)
**Pathological features**	
Grading in biopsy	
G1	211 (46.2)
G2	18 (4.2)
No grading possible/no biopsy performed	200 (46.6)
Grading changes (biopsy → surgical specimen)	
Any changes in grading	85 (19.8)
Upgrade G1 → G2	59 (13.8) 59/85 (69.4)
Upgrade G2 → G3	6 (1.4) 6/85 (7.1)
Downgrade G2 → G1	18 (4.2) 18/85 (21.2)
Downgrade G1 → Non-invasive	2 (0.5) 2/85 (2.4)
Subtypes in biopsy	364 (84.8)
NST	187/364 (51.4)
Pure special type	23/364 (6.3)
Cribriform	8/364 (2.2) 8/23 (34.8)
Lobular	3/364 (0.8) 3/23 (13)
Mucinous	3/364 (0.8) 3/23 (13)
Papillary	3/364 (0.8) 3/23 (13)
Tubular	6/364 (1.7) 6/23 (26.1)
Mixed subtype (>1 subtype)	23/364 (6.3)
Non-invasive histology	41/364 (11.3)
No biopsy performed/available	90/364 (24.7)

**Table 2:** Patients with G1 carcinoma in surgical specimens (*n* = 364).

	***n* (%)**
**Patients’ characteristics**	
Sex	
Female	364 (100)
Male	0 (0)
Age at diagnosis (years)	
median (IQR; range)	62 (52–71; 33 to 92)
≤50	75 (20.6)
>50	289 (79.4)
**Pathological features**	
Grading in surgical specimen	
G1	364 (100)
Subtypes in surgical specimen	
NST	245 (67.3)
Pure special type	55 (15.1)
Cribriform	11 (3) 11/55 (20)
Lobular	4 (1.1) 4/55 (7.3)
Mucinous	13 (3.6) 13/55 (23.6)
Papillary	6 (1.6) 6/55 (10.9)
Tubular	20 (5.5) 20/55 (36.4)
Adenoid cystic	1 (0.3) 1/55 (1.8)
Mixed subtype (>1 subtype)	57 (15.7)
Non-invasive histology (DCIS)	7 (2)
Nodal stage	
pN0	244 (67)
pN1	40 (11)
pN1mi (sn)	46 (12.6)
pN2	10 (2.8)
pN3	3 (0.8)
pNX or not available	21 (5.8)
Tumour stage	
pT1	294 (80.8)
pT2	62 (17)
pT3	1 (0.3)
pT4	1 (0.3)
rpT1	2 (0.6)
rpT2	1 (0.3)
rpT4b	1 (0.3)
pTX or not available	2 (0.6)
M stage	
M0	348 (95.6)
M1	1 (0.3)
MX	15 (4.1)
ER status	
ER strongly positive (>10%)	350 (96.2)
ER low positive (1–9%)	3 (0.8)
ER negative (0%)	1 (0.3)
Not available	10 (2.8)
PR status	
PR strongly positive (>10%)	315 (86.5)
PR low positive (1–9%)	17 (4.7)
PR negative (0%)	19 (5.2)
Not available	13 (3.6)
Her2 status	
HER positive (FISH+ and/or IHC 3+)	1 (0.3)
HER negative (FISH- and/or IHC 0/1+/2+)	352 (96.7)
Not available	11 (3)
**Events**	
Patients with events	31 (8.5)
Ipsilateral recurrence (same breast/axilla)	10 (2.8) 10/31 (32.3)
Second cancer/contralateral recurrence	15 (4.1) 15/31 (48.4)
Distant metastasis	6 (1.7) 6 (1.7)

**Table 3:** Mixed subtypes diagnosed in surgical specimens.

	***n* (%)**
**Mixed subtypes in surgical specimens**	
NST + cribriform	18 (31.6)
NST + tubular	9 (15.8)
NST + papillary	1 (1.8)
NST + mucinous	3 (5.3)
NST + lobular	4 (7)
NST + micropapillary	2 (3.5)
Tubular + lobular	1 (1.8)
Tubular + cribriform	1 (1.8)
NST + cribriform + tubular	9 (15.8)
NST + cribriform + lobular	1 (1.8)
NST + cribriform + papillary	2 (3.5)
NST + cribriform + encapsulated non-invasive papillary carcinoma	1 (1.8)
NST + tubular + lobular	2 (3.5)
Tubular + lobular + papillary	1 (1.8)
Tubular + lobular + cribriform	1 (1.8)
NST + cribriform + lobular + tubular	1 (1.8)
Total	57 (100)

## Data Availability

The original contributions presented in this study are included in the article/Supplementary Materials. Further inquiries can be directed to the corresponding author.

## Supplementary Materials

The following supporting information can be downloaded at https://www.mdpi.com/article/10.3390/cancers18193154/s1: Figure S1: Distribution of subtypes of G1 carcinomas (NST vs. pure special type vs. mixed subtypes) in surgical specimens between 2005–2014; Figure S2: Kaplan–Meier survival curves in pure special subtypes of G1 carcinomas in surgical specimens in a follow-up period of 20 years. OP: Operation. There is no significant difference in overall survival stratified to special histological subtype (*p* = 0.2); Table S1: Operations per year; Table S2: Survival data in 348 of 364 patients (only 348 patients were included in the survival analysis).
