# Supplementary material for: Histological Subtype Provides Limited Prognostic Information in Grade 1 Invasive Breast Carcinoma: A Retrospective Cohort Study
Source: Cancers (Basel). 2026 Sep 29;18(19):3154. doi: 10.3390/cancers18193154 (PMC13664015; doi:10.3390/cancers18193154)
Supplement: Supplementary file 1 [file cancers-18-03154-s001.zip › cancers-4513671-supplementary.pdf]

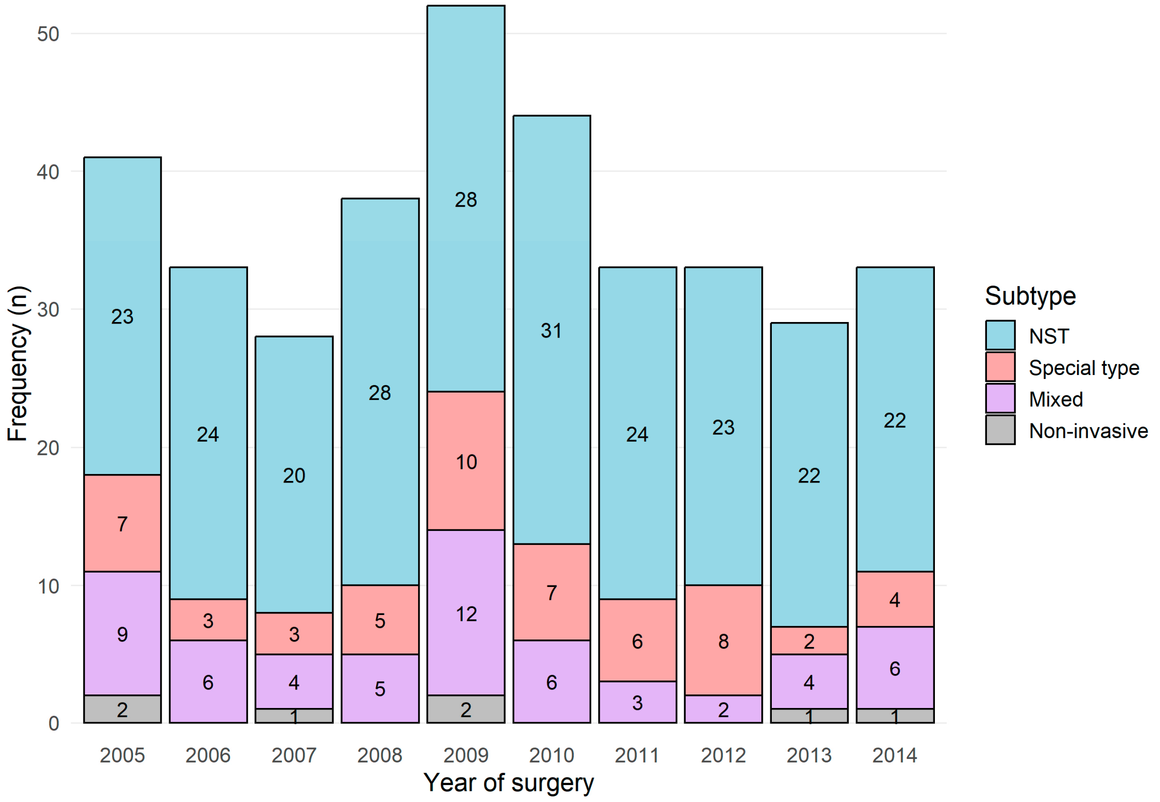

Figure S1. Distribution of subtypes of G1 carcinomas (NST vs pure special type vs mixed subtypes) in surgical specimens between 2005-2014.

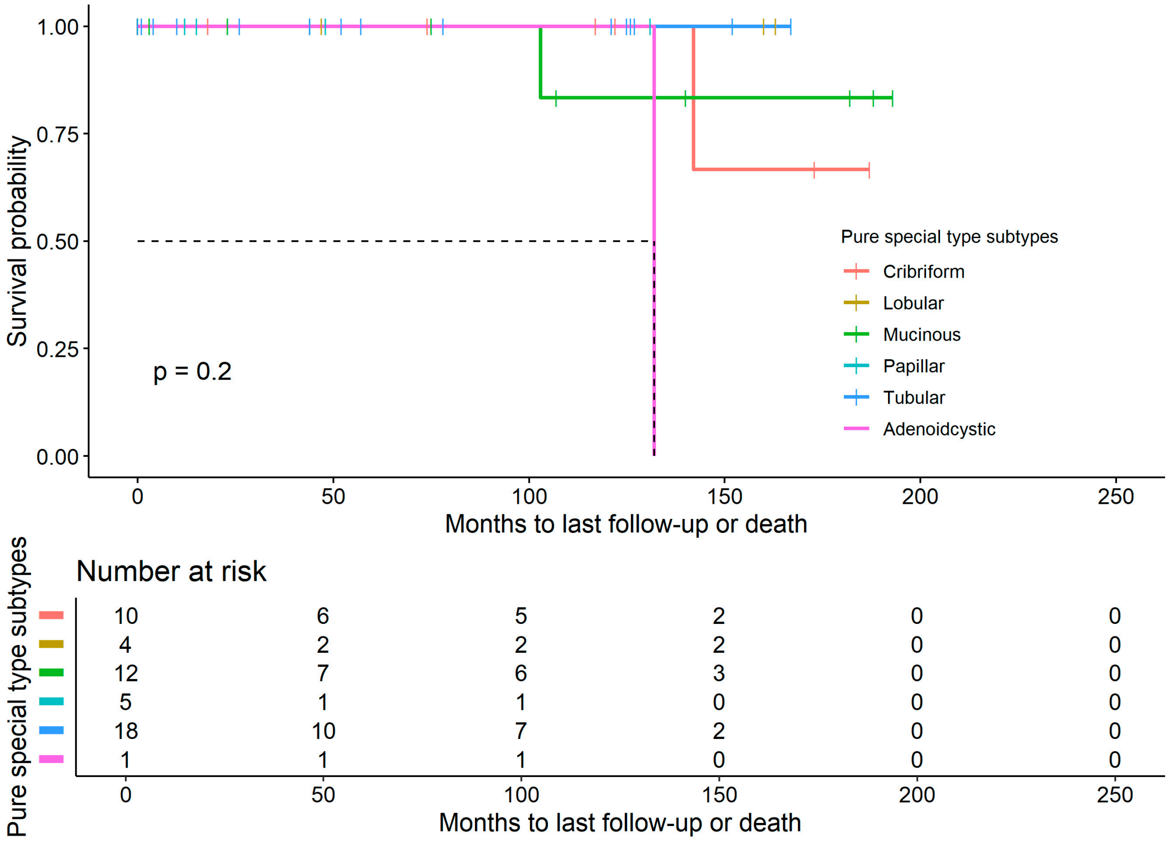

Figure S2. Kaplan-Meier survival curves in pure special subtypes of G1 carcinomas in surgical specimens in a follow-up period of 20 years. OP: Operation. There is no significant difference in overall survival stratified to special histological subtype (p=0.2).

Table S1. Operations per year.

	**n (%)**
**Operations per year (n=364)**	
2005	41 (11.3)
2006	33 (9.1)
2007	28 (7.7)
2008	38(10.4)
2009	52 (14.3)
2010	44 (12.1)
2011	33 (9.1)
2012	33 (9.1)
2013	29 (8)
2014	33 (9.1)
Total	364 (100)

Table S2. Survival data in 348 of 364 patients (only 348 patients were included in the survival analysis).

	**Overall**	**NST**	**Pure special type**	**Mixed subtype**	**Non-invasive**
Patients (n)	348	239	50	52	7
5-year survival rate (%)	94.7	94.1	100	92.2	100
10-year survival rate (%)	85.4	84.7	95.5	79.5	100
Deaths (n (%))	46 (13.2)	32 (13.4)	3 (6)	11 (21.2)	0 (0)
Censoring (n (%))	302 (86.8)	207 (86.6)	47 (94)	41 (78.8)	7 (100)
